# The putative protein methyltransferase LAE1 controls cellulase gene expression in *Trichoderma reesei*

**DOI:** 10.1111/j.1365-2958.2012.08083.x

**Published:** 2012-06

**Authors:** Bernhard Seiboth, Razieh Aghcheh Karimi, Pallavi A Phatale, Rita Linke, Lukas Hartl, Dominik G Sauer, Kristina M Smith, Scott E Baker, Michael Freitag, Christian P Kubicek

**Affiliations:** 1 Institute of Chemical Engineering, University of Technology of ViennaGumpendorferstrasse 1a, A-1060 Vienna, Austria; 2 Department of Biochemistry and Biophysics, Center for Genome Research and Biocomputing, Oregon State UniversityCorvallis, OR 97331, USA; 3 Austrian Center of Industrial Biotechnology (ACIB), c/o Institute of Chemical Engineering, University of Technology of ViennaGumpendorferstrasse 1a, A-1060 Vienna, Austria; 4 Fungal Biotechnology Team, Chemical and Biological Process Development Group, Pacific Northwest National Laboratory902 Battelle Blvd., Richland, WA 99352, USA

## Abstract

*Trichoderma reesei* is an industrial producer of enzymes that degrade lignocellulosic polysaccharides to soluble monomers, which can be fermented to biofuels. Here we show that the expression of genes for lignocellulose degradation are controlled by the orthologous *T. reesei* protein methyltransferase LAE1. In a *lae1* deletion mutant we observed a complete loss of expression of all seven cellulases, auxiliary factors for cellulose degradation, β-glucosidases and xylanases were no longer expressed. Conversely, enhanced expression of *lae1* resulted in significantly increased cellulase gene transcription. *Lae1*-modulated cellulase gene expression was dependent on the function of the general cellulase regulator XYR1, but also *xyr1* expression was LAE1-dependent. LAE1 was also essential for conidiation of *T. reesei*. Chromatin immunoprecipitation followed by high-throughput sequencing (‘ChIP-seq’) showed that *lae1* expression was not obviously correlated with H3K4 di- or trimethylation (indicative of active transcription) or H3K9 trimethylation (typical for heterochromatin regions) in CAZyme coding regions, suggesting that LAE1 does not affect CAZyme gene expression by directly modulating H3K4 or H3K9 methylation. Our data demonstrate that the putative protein methyltransferase LAE1 is essential for cellulase gene expression in *T. reesei* through mechanisms that remain to be identified.

## Introduction

The β-(1,4)-linked glucose polymer cellulose and hemicellulose polysaccharides of varying composition make up 60–80% of the plant cell wall and arise from the utilization of solar energy and carbon dioxide by plants at an annual production rate of about 7.2 and 6 × 10^10^ tons respectively (Gilbert, 2010). These polymers make up a significant portion of the total plant biomass, and degradation of these polysaccharides is a key transformation step in the biological carbon cycle in nature.

Most industrial production of enzymes for plant biomass hydrolysis is performed with mutants of the fungus *Trichoderma reesei* (the anamorph of the tropical ascomycete *Hypocrea jecorina*) (Xu *et al*., 2009). Consequently, this fungus serves as the model system for the molecular understanding of cellulase gene expression and secretion of the encoded cellulase proteins. To this end, its genome has recently been sequenced (Martinez *et al*., 2008). Interestingly, *T. reesei* contains a lower number of genes coding for cellulases and hemicellulases when compared with genomes of other ascomycete fungi, such as *Podospora anserina, Gibberella zeae* or *Magnaporthe grisea* (Espagne *et al*., 2008). Moreover, in contrast to all other ascomycetes whose genome has been sequenced*, T. reesei* cellulase, hemicellulase and other carbohydrate-active enzyme (CAZyme) encoding genes were found to be fivefold enriched in several discrete clusters. Regions of high CAZyme gene density also contain genes encoding secondary metabolic enzymes, such as non-ribosomal polypeptide synthases (NRPS) and polyketide synthases (PKS) (Martinez *et al*., 2008).

Genes encoding enzymes involved in the biosynthesis of secondary metabolites are known to occur in clusters, often near the telomeres of chromosomes (Keller and Hohn, 1997). In the ascomycete genus *Aspergillus*, such clusters of secondary metabolite genes are proposed to be regulated at the level of histones by the putative protein methyltransferase LaeA (Bok and Keller, 2004; Bayram *et al*., 2008), which somehow reverses gene repression at the level of heterochromatin structure (Reyes-Dominguez *et al*., 2010; Strauss and Reyes-Dominguez, 2011). Because of the co-clustering of genes for cellulases, hemicellulases and other CAZymes with those for secondary metabolite synthesis in the *T. reesei* genome, we hypothesized that cellulase expression may be regulated by a *T. reesei* LaeA orthologue.

Here we describe the identification of a LaeA orthologue in *T. reesei*, LAE1. Furthermore, we show that manipulation of its expression has a dramatic effect on cellulase and hemicellulase gene expression. However, this phenotype is not associated with alterations in H3K4 or H3K9 methylation at cellulase and hemicellulase loci, suggesting that the effect on gene expression is indirect.

## Results

### Identification of the *T. reesei* LAE1 orthologue

To identify *lae1*, we first screened the 92 predicted *S*-adenosylmethionine-dependent methyltransferases in the *T. reesei* genome database (http://genome.jgi-psf.org/Trire2/Trire2.home.html). When any of the functionally verified *Aspergillus* LaeA proteins (Bok *et al*., 2005; Bayram *et al*., 2008) were used as a query in blastp, psi-blast or phi-blast, several high scoring hits were obtained. However, when reciprocal queries were subsequently made against the *Aspergillus* genome databases (http://www.broadinstitute.org/annotation/genome/aspergillus_group/MultiHome.html), the LaeA orthologues used were not obviously identified. Since this approach therefore led to potential false positives, we used an iterative phylogenetic strategy to identify the *T. reesei* LaeA orthologue. We used blastp to detect LaeA orthologues in fungal species more closely related to the *Aspergilli* (such as *Coccidioides immitis*), then used the identified proteins to look for LaeA orthologues in *Dothidiomycetes*, and used the latter one to the *Sordariomycetes* and finally the *Hypocreaceae*. By this method we arrived at 27 putative LaeA orthologues from *Eurotiomycetes*, *Dothidiomycetes* and *Sordariomycetes*.

In order to determine the correct amino acid sequence of the most likely candidate for the *T. reesei* LAE1 protein (Trire2:41617), its cDNA was sequenced, which led to the identification of two additional introns and one exon than predicted from the *T. reesei* genome database and an increased similarity to other fungal LaeA/LAE1 proteins. The GenBank accession number for the cDNA is JN791097.

**Figure 1:**
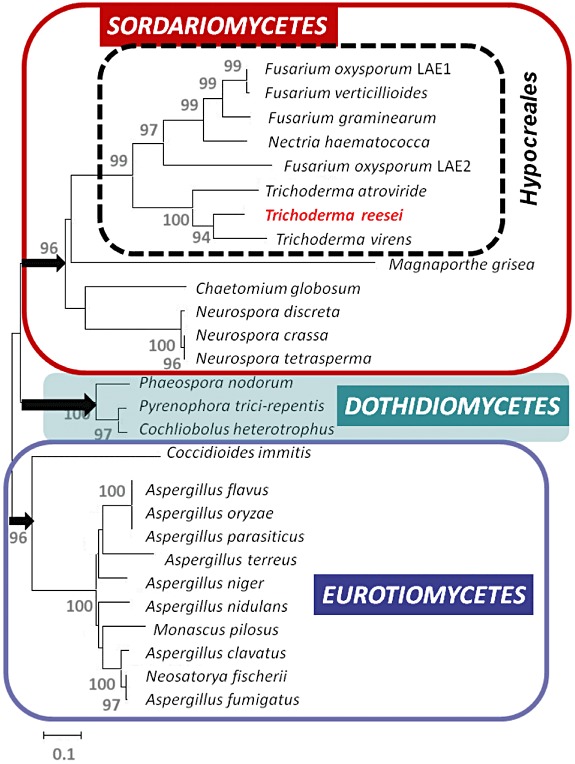
Phylogenetic analysis of LaeA/LAE1 proteins from *Eurotiomycetes*, *Dothidiomycetes* and *Sordariomycetes.* Accession numbers for protein sequences are listed in Table S3. The tree was constructed by Neighbor Joining in MEGA 5.0 (Tamura *et al*., 2011) with 500 bootstrap replicates (coefficients are indicated below the respective nodes). Gaps in the alignment were not considered.

Phylogenetic analysis of LaeA protein sequences (Fig. 1) produced a tree whose branching was consistent with established phylogenetic relationships between the various taxa, suggesting orthology of the identified protein sequences. Thus, we consider Trire2:41617 the *T. reesei* LaeA orthologue, which we named LAE1.

### *lae1* is essential for cellulase gene expression in *T. reesei*

**Figure 2:**
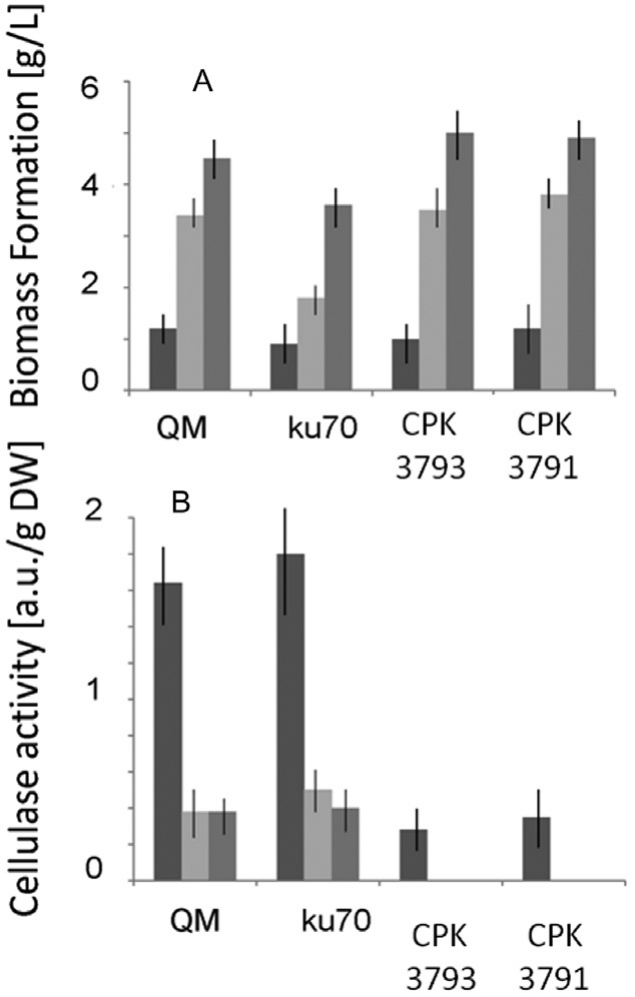
Effect of loss-of-function of *lae1* on biomass formation and cellulase/hemicellulase enzyme formation by *T. reesei.* Growth (A) and cellulase formation (B) of *T. reesei* QM 9414, the transformation recipient *ku70* and the corresponding Δ*lae1* strains CPK3793 and CPK3791 on 1% (w/v) lactose. Cellulase expression is given in arbitrary units and related to the respective biomass dry weight of the strain at the respective time point (given in A). The three bars represent (from left to right) values for 48, 72 and 96 h of cultivation. Experiments are means of three biological replicates, and the SD given by vertical bars.

To investigate a possible impact of *lae1* on cellulase production in *T. reesei*, *lae1* null mutants (Δ*lae1*) of the moderate cellulase producing mutant strain *T. reesei* QM 9414 were generated by replacing the *lae1* coding region with the orotidine-5-decarboxylase gene *pyr4* in a KU70-deficient (Δ*tku70*) strain (Guangtao *et al*., 2009). Since retransformation of the latter strain was not possible, we investigated the phenotype of several deletion mutants. Their growth on simple carbon sources such as glycerol was similar to that of the parent strain but growth on cellulose was severely impaired (data not shown), implying that *lae1* is required for normal growth on cellulose. To test whether this was caused by a loss of cellulase production, we cultivated the *lae1* deletion strains and the parent strain on lactose, a carbon source that induces cellulase expression, but whose utilization is independent of the action of secreted cellulases (Seiboth *et al*., 2007). Growth of the parent strains *T. reesei* QM 9414 and ku70 was indeed similar (Fig. 2A), but significantly reduced cellulase activities were found in the cultures of the Δ*lae1* strain (Fig. 2B). Similar findings were also obtained with xylan as a carbon source, on which Δ*lae1* mutants exhibited strongly reduced xylanase activity (data not shown).

### *lae1* regulates expression of the CAZyme gene clusters in *T. reesei*

**Figure 3:**
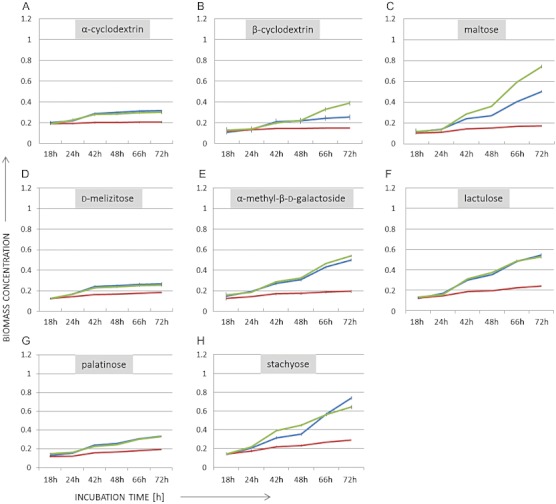
Growth of *T. reesei* QM 9414 (blue), the Δ*lae1* strain (CPK3793, red) and the strain expressing *tef1:lae1* (CPK4086, green) on several oligosaccharides. Data were obtained from Phenotype microarrays as described (Druzhinina *et al*., 2006). Carbon sources used were: (A) α-cyclodextrin, (B) β-cyclodextrin, (C) maltose, (D) d-melizitose [α-d-glucopyranosyl-(1→3)-*O*-β-d-fructofuranosyl-(2→1)-α-d-glucopyranoside], (E) α-methyl-β-d-galactoside, (F) lactulose [4-*O*-*β*-d-galactopyranosyl-d-fructofuranose], (G) palatinose [6-*O*-α-d-Glucopyranosyl-d-fructose], (H) stachyose [β-d-Fructofuranosyl-*O*-α-d-galactopyranosyl-(1→6)-*O*-α-d-galactopyranosyl-(1→6)-α-d-glucopyranoside]. The vertical axis shows the OD_750_ that is equivalent to biomass formation (g l^−1^).

Results described above provided evidence of LAE1 influence on cellulase and hemicellulase expression by *T. reesei*; however, these effects cannot be validated solely from growth data. In order to test whether deletion of *lae1* indeed impairs the expression of biomass-degrading enzymes, we carried out transcriptome analysis. Microarrays representing all 9143 unique predicted ORFs in the *T. reesei* genome were used to determine their relative expression in the wild-type and Δ*lae1* strains when grown on lactose as a carbon source**.** We found 769 genes with at least a twofold decrease in their hybridization intensity in the Δ*lae1* strain compared with the control, QM 9414. Among these, 50 CAZyme-encoding genes were detected, the majority of which comprised glycosyl hydrolases (GH) involved in cellulose and hemicellulose degradation (Table 1; see also Table S1). This included 9 of the 10 cellulases and cellulase-enhancing proteins (CEL61) present in *T. reesei*; only the cellulase-monooxygenase CEL61C (TRire2:27554) was absent from this list. Transcripts for other proteins were also downregulated, such as the swollenin SWO1, a protein carrying an expansin-like domain that disrupts the crystalline cellulose structure (Saloheimo *et al*., 2002), CIP1, which contains a signal peptide and a cellulose-binding domain (Foreman *et al*., 2003; Li *et al*., 2007), and four xylanases (XYN1 to XYN4). These findings imply that expression of most of the cellulolytic and hemicellulolytic genes is affected by deletion of *lae1*. The majority of the other affected GHs (21 of 28) comprised glycosidases active against various side-chains in hemicelluloses. The reduction of expression of various glycosidase genes was also reflected in the decreased ability to grow on several of their di- or oligosaccharide substrates (Fig. 3).

**Table 1:** Changes in CAZome gene expression in *T. reesei* after deletion of *lae1*.^a^

	Protein ID	Downregulated	*P*-value

**Cellulases**			
GH 5 endo-β-1,4-glucanase CEL5A	120312	17.338	0.000896
GH5 endo-β-1,4-glucanase CEL5B	82616	5.179	0.00219
GH6 Cellobiohydrolase 2 CEL6A	72567	15.565	0.00106
GH7 endo-β,4-glucanase CEL7B	122081	3.019	0.00126
GH7 cellobiohydrolase 1 CEL7A	123989	6.841	0.000994
GH12 endo-β-1,4-glucanase CEL12A	123232	15.789	0.00132
GH45 endo-β-1,4-glucanase CEL45	49976	14.409	0.000892
GH61 cellulase enhancing protein CEL61A	73643	25.659	0.000851
GH61 cellulase enhancing protein CEL61B	120961	40.524	0.000836
GH1 β-glucosidase CEL1B	22197	3.454	0.000918
GH1 β-glucosidase CEL1A	120749	2.428	0.00107
GH3 β-glycosidase of uncertain specificity	108671	2.833	0.00142
GH3 β-glucosidase CEL3D	46816	2.115	0.00525
GH3 β-glucosidase CEL3C	82227	3.555	0.00153
**Non-enzymatic cellulose attacking enzymes**			
CIP1	73638	16.794	0.000855
CBM13 protein	111094	8.529	0.00407
Swollenin	123992	3.714	0.000847
Swollenin-like, 84% ID to 123992	111874	6.17	0.00153
**Xylanases**			
GH10 xylanase XYN3	120229	4.392	0.00202
GH11 xylanase XYN1	74223	2.038	0.00213
GH11 xylanase XYN2	123818	23.487	0.000931
GH30 xylanase XYN4	111849	2.121	0.000885
GH3 β-xylosidase BXL1	121127	17.003	0.000896
GH43 β-xylosidase/α-arabinofuranosidase	3739	2.752	0.000911
GH74 xyloglucananase CEL74A	49081	3.051	0.0009
**Hemicellulose side-chain cleaving enzymes**			
CE5 acetyl xylan esterase AXE1	73632	6.821	0.000951
GH67 α-glucuronidase AGU1	72526	13.365	0.00101
CIP2 methyl glucuronoyl esterase	123940	3.232	0.00118
GH54, l-α-arabinofuranosidase ABF1	55319	2.201	0.00158
GH95 α-fucosidase	58802	9.946	0.00544
GH95 α-fucosidase	5807	3.606	0.0018
GH92 α-1,2-mannosidase	74198	6.097	0.000712
GH92 α-1,2-mannosidase	60635	2.154	0.00372
GH47 α-1,2-mannosidase	45717	4.496	0.00133
GH2 β-mannosidase	69245	5.937	0.00181
GH27 α-galactosidase AGL1	72632	5.777	0.00134
GH27 α-galactosidase AGL3	27259	2.065	0.00641
**Pectinases**			
GH28 polygalacturonase	103049	2.382	0.00831

a Values are given as means of two biological replicates; ‘downregulation’ is given as -fold decrease; CPK3793 was used as *lae1* delta strain.

The 25 CAZyme gene clusters in the *T. reesei* genome contain an average fivefold increase in CAZyme gene density compared with the expected density for randomly distributed genes (Martinez *et al*., 2008). In total, 126 of the approximately 320 CAZyme genes are found in these regions ranging from 14 kb to 275 kb in length. As we identified 769 of the total 9143 genes in the *T. reesei* genome, we would expect one gene with decreased gene expression at every twelfth locus if their distribution would be random. However, if the genes are clustered as calculated above, those with decreased expression should occur in at least every third locus or even be closer to each other. To investigate this, we mapped the 769 identified genes onto the *T. reesei* scaffolds and searched for potential clustering. Indeed, we found 28 regions on 21 scaffolds that exhibited an at least threefold increase of genes with changed expression over the random distribution (Table 2). The null hypothesis of random distribution of the clusters was rejected because of the low Pearson coefficient (*r* = 0.04; *t* = 0.15). On average, these clusters comprised 7.2 genes and were 6.6-fold enriched over the average. Interestingly, 13 of these 28 clusters were found in areas not previously predicted as CAZyme gene clusters (Martinez *et al*., 2008).

**Table 2:** Clusters of expressed genes affected in *T. reesei*Δ*lae1*.

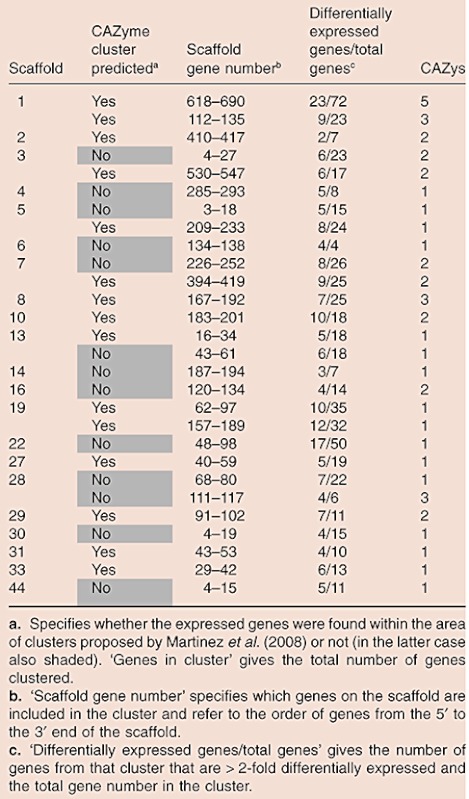

### LAE1 dependence of cellulase gene expression is inducer independent

**Figure 4:**
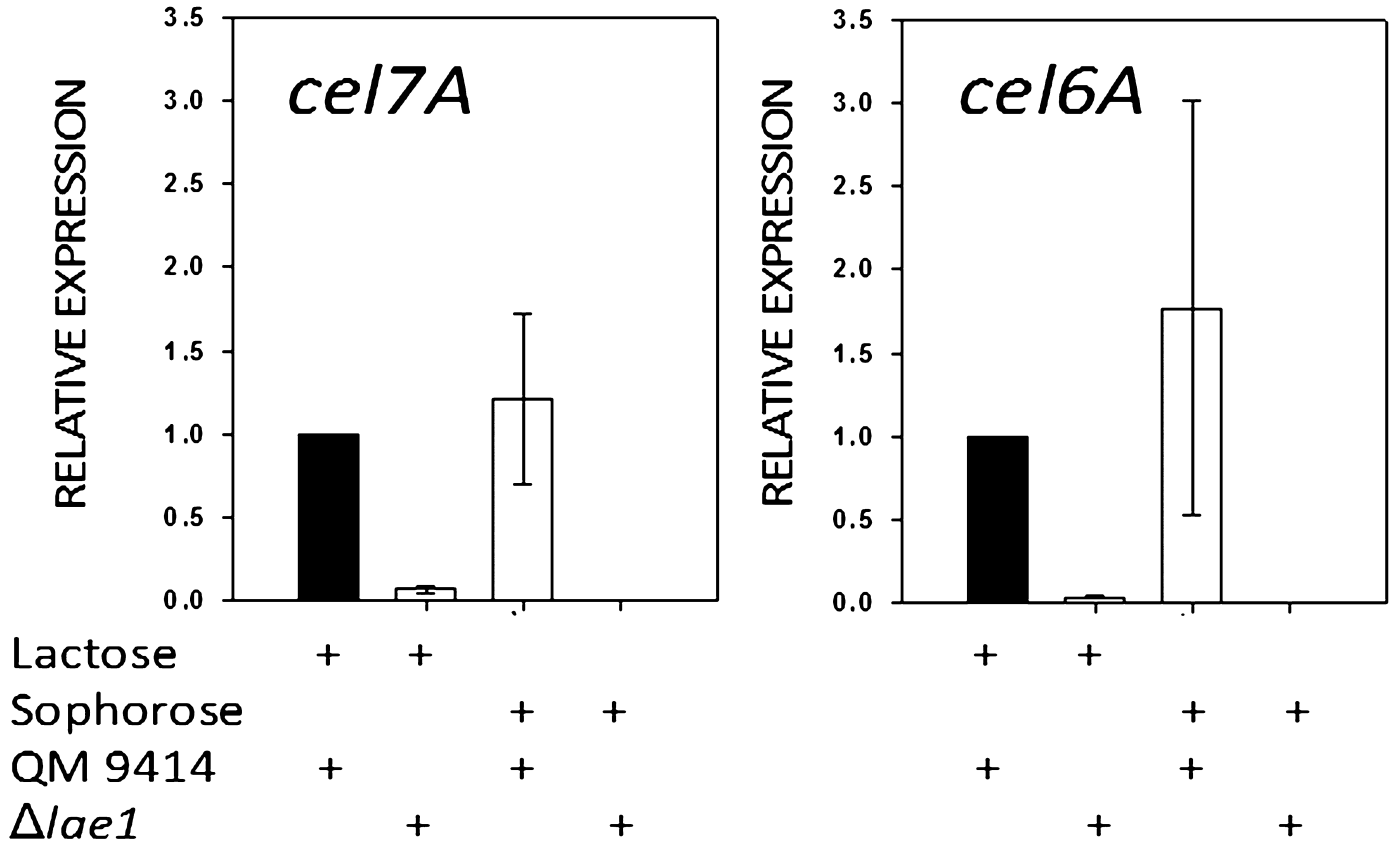
Expression of the two cellobiohydrolase-encoding genes *cel7a* and *cel6a* in *T. reesei* QM 9414 and the Δ*lae1* strain CPK3793 during growth on lactose or incubation with sophorose. Cellulase transcript levels in QM 9414 during growth on lactose are given with full bars and set to 1.0. The respective transcript levels in relation to QM 9414 are shown with open bars. Data are means of triplicate determinations from two biological replicates.

In order to confirm the results from microarray analysis, we used *cel7A* and *cel6A* as cellulase model genes and tested their expression in the parent strain and in the Δ*lae1* strain by qRT-PCR. We used cultures grown on lactose or induced with sophorose, a disaccharide conferring high cellulase induction in resting cells (Sternberg and Mandels, 1979) (Fig. 4). qRT-PCR data confirmed the results from the microarray experiments, as cellulase gene expression on the two soluble carbon sources was absent in the Δ*lae1* strains. The results also demonstrate that cellulase regulation by LAE1 is independent of the nature of the inducer.

### Introducing a constitutively expressed *lae1* allele into *T. reesei* enhances cellulase formation

**Figure 5:**
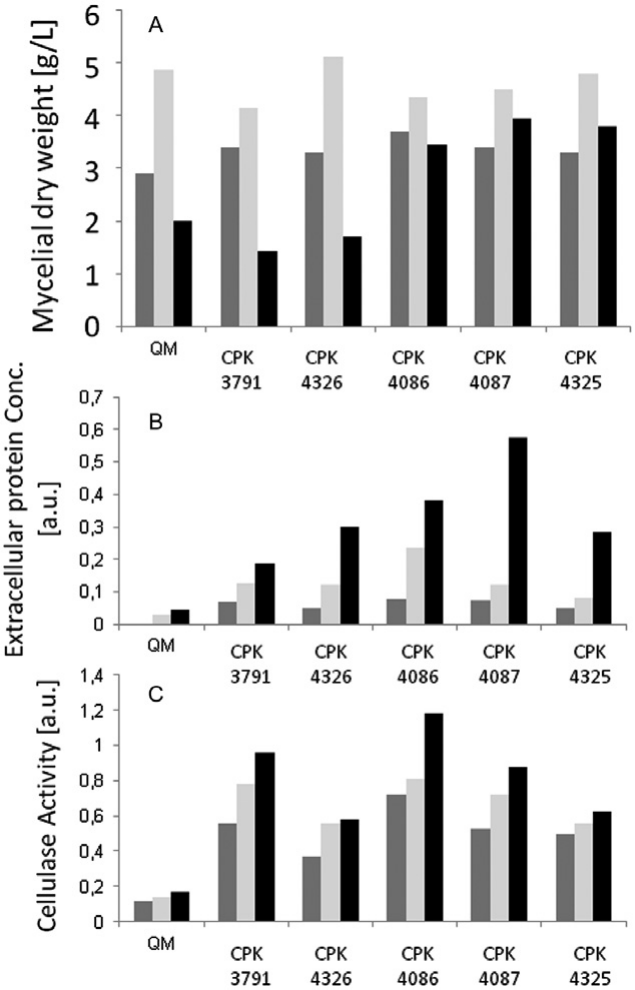
Biomass formation (A), cellulase production (B) and extracellular protein (C) during growth of *T. reesei* QM 9414 (QM) and several mutant strains bearing additional copies of the *tef1:lae1* gene construct (CPK3791, CPK4326, CPK4086, CPK4087, CPK4325) on lactose. The three bars represent (from left to right) values for 48, 72 and 96 h of cultivation. Each bar is from a single experiment only but representative of at least four biological replica that were consistent with the claims.

**Figure 6:**
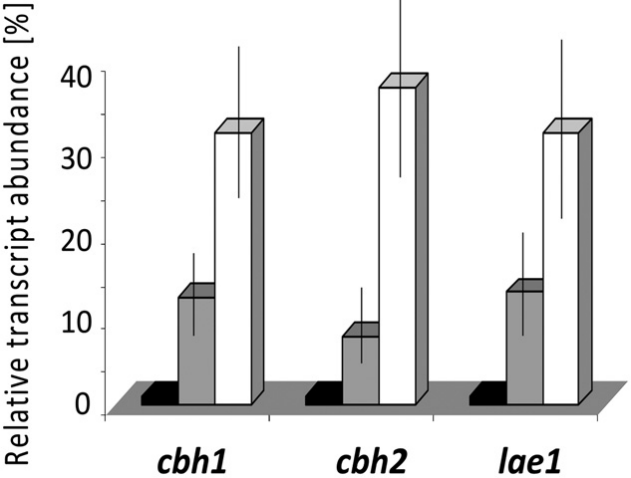
Relative abundance of transcripts for *cel7a, cel6a* and *lae1* at 26 h of growth on lactose in two *T. reesei tef1:lae1* mutant strains (CPK4086, grey bars; CPK3791, white bars) in relation to the QM 9414 recipient strain (black bars). Transcripts were normalized to the housekeeping gene *tef1*, and the respective ratio in QM 9414 set to 1. Other strains are given in percentage to that of the QM 9414. Copy numbers were determined as described in *Experimental procedures*: CPK4086 had two additional copies indicating a single ectopically integrated copy; CPK3791, however, had three additional copies, suggesting more than one ectopically integrated copy, but the exact number was not determined. Vertical bars indicate SD.

**Figure 7:**
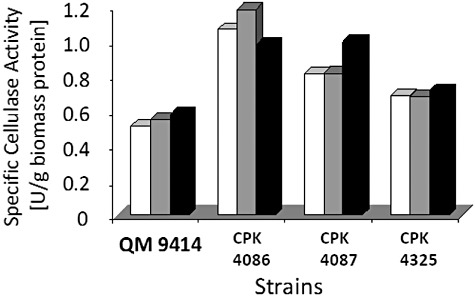
Cellulase activity during growth of selected *T. reesei tef1:lae1* mutant strains on cellulose. Activities are given as arbitrary units and are related to 1 g of fungal biomass protein. Bars indicate measurements after 7, 9 and 11 days of incubation (from left to right). Data are means from four measurements and two independent biological replicates.

Having identified LAE1 as a regulator of cellulase and hemicellulase biosynthesis in *T. reesei*, we hypothesized that its activity in the parent strain could be limiting for cellulase gene expression. We therefore tested whether increased activity of LAE1 by overexpression of *lae1* would stimulate cellulase formation in the same strain. We fused the *lae1* ORF to the 5′-upstream sequences of the *tef1* (translation elongation factor 1α-encoding) gene, which was expected to result in high constitutive expression of *lae1*. Three *T. reesei* strains that contained either one or two copies of the *tef1:lae1* construct integrated ectopically in the genome were examined for their ability to produce cellulases on lactose. Growth of all transformants was comparable until 72 h of cultivation, but – unlike the wild-type strain – several of them did not start to autolyse thereafter (indicated by the loss in mycelia dry weight; Fig. 5A). The strains bearing the *tef1:lae1* copies exhibited up to 10-fold increased protein and cellulase formation, which was highest in the strains that did not show autolysis (Fig. 5B and C). We also quantified the expression of *cel7A* and *cel6A* and found them to be 10- to 40-fold increased (Fig. 6). This increased expression correlated with a five- to eightfold increased expression of *lae1* in these strains. The higher *lae1* expression in CPK3791 may be due to the presence of more than one ectopically integrated *lae1* copy (as in CPK4086) but the exact copy number was not determined. Growth of the *tef1:lae1* mutant strains on cellulose also confirmed increased cellulase formation, although less dramatically than on lactose (Fig. 7). The comparatively smaller effect of *lae1* overexpression on cellulose may be due to the fact that cellulases are required for growth, and their overexpression may lead to an excess of cellobiose and glucose formed that in turn induces carbon catabolite repression of cellulase expression.

**Figure 8:**
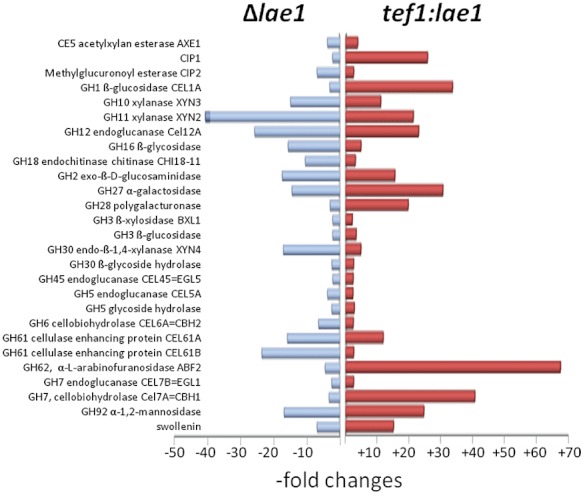
Relative changes in expression of CAZome genes in the Δ*lae1* (CPK3793) and the *tef1:lae1* (CPK4086) strains, given as the fold changes of transcript hybridization in relation to the parent strain. Only values with *P* < 0.05 are shown.

Microarray expression analysis revealed 68 CAZyme genes to be significantly upregulated (> 2-fold) in the *tef1:lae1* strain CPK4086 (Table S1). A comparison with the CAZyme genes that were downregulated in the Δ*lae1* strain showed that 27 genes were consistently affected in both strains, i.e. exhibited strongly reduced expression in the Δ*lae1* strain but enhanced expression in the *tef1:lae1* strain (Fig. 8). These genes included eight of the 10 cellulases and cellulase-enhancing proteins (see above), the xylanases XYN1, XYN2, XYN3 and XYN4, and the auxiliary factors CIP1 and swollenin. The remaining genes comprised various β-glycosidases active on hemicelluloses side-chains. Interestingly, CAZyme genes that responded only to *lae1* overexpression included eight chitinases, six β-glucanases and two CBM13 carbohydrate binding modules, suggesting that *lae1* regulation of CAZyme gene expression may play roles beyond cellulose degradation.

### Regulation of expression of cellulases by *lae1* is dependent on the function of XYR1

**Figure 9:**
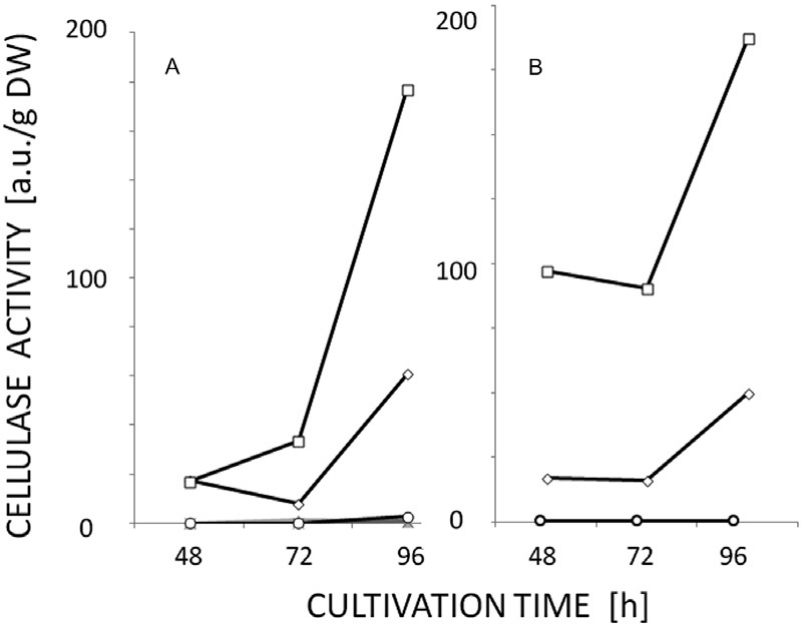
Cellulase formation in delta-*lae1* (CPK3793) and delta-*xyr1* strains of *T. reesei* is unaffected by the constitutive overexpression of *xyr1* or *lae1*. A. Overexpression of *xyr1* in a delta-*lae1* background: three different transformants yielded identical results and thus shown only for one of them (circles). The wild-type QM 9414, and QM 9414 containing a single *pki1:xyr1* copy are given as comparison (diamonds and squares respectively). B. Overexpression of *lae1* in a delta-*xyr1* background: three different transformants yielded again identical results and thus shown only for one of them (circles). The wild-type QM 9414, and strain CPK4086 overexpressing *tef1:lae1* are given as comparison (diamonds and squares respectively). Data from a single experiment only are shown, but are consistent with the results from at least two separate biological replicas.

The above data suggest that regulation of cellulase and hemicellulase gene expression cannot bypass the necessity of an inducer. In order to investigate this at a molecular level, we replaced the *xyr1* gene, encoding the major cellulase and hemicellulase regulator XYR1 (Stricker *et al*., 2006), by a constitutively expressed *pki1:xyr1* allele in the *Δlae1* strain. Likewise, we deleted *xyr1* in the *lae*1OE strain. The data, shown in Fig. 9, demonstrate that neither the overexpression of *lae1* in a *Δxyr1* background, nor the overexpression of *xyr1* in a delta-*lae1* background resulted in cellulase formation. Increased transcription of *xyr1* and *lae1* from the constitutive promoters was proven by qPCR (data not shown). These data are consistent with the inducer dependence of *lae1* overexpression, and demonstrate that *lae1* requires the function of *xyr1*, but also that *xyr1* depends on the function of *lae1*.

### Expression of CAZyme genes in *Δlae1* and *tef1:lae1* is not correlated with methylation at histone H3 lysine 4 or lysine 9

Expression of secondary metabolism gene clusters in *Aspergillus* is affected by LaeA (Bok and Keller, 2004; Bayram *et al*., 2008), presumably by changing specific histone modifications (see review by Strauss and Reyes-Dominguez, 2011). Direct biochemical evidence for this hypothesis is still lacking. To test this hypothesis for regulation of the CAZyme genes in *T. reesei*, we performed chromatin immunoprecipitation followed by high-throughput sequencing (‘ChIP-seq’) on wild type, Δ*lae1* and *tef1:lae1* strains that had been grown in lactose containing medium for 26 h. Histone H3 lysine 4 (H3K4) dimethylation is indicative of the potential for transcription and H3K4 trimethylation (H3K4me3) suggests active transcription of genes associated with nucleosomes that carry this modification (Hublitz *et al*., 2009). H3K9me3 is associated with gene silencing, typically in regions of facultative or constitutive heterochromatin (Lewis *et al*., 2009).

**Figure 10:**
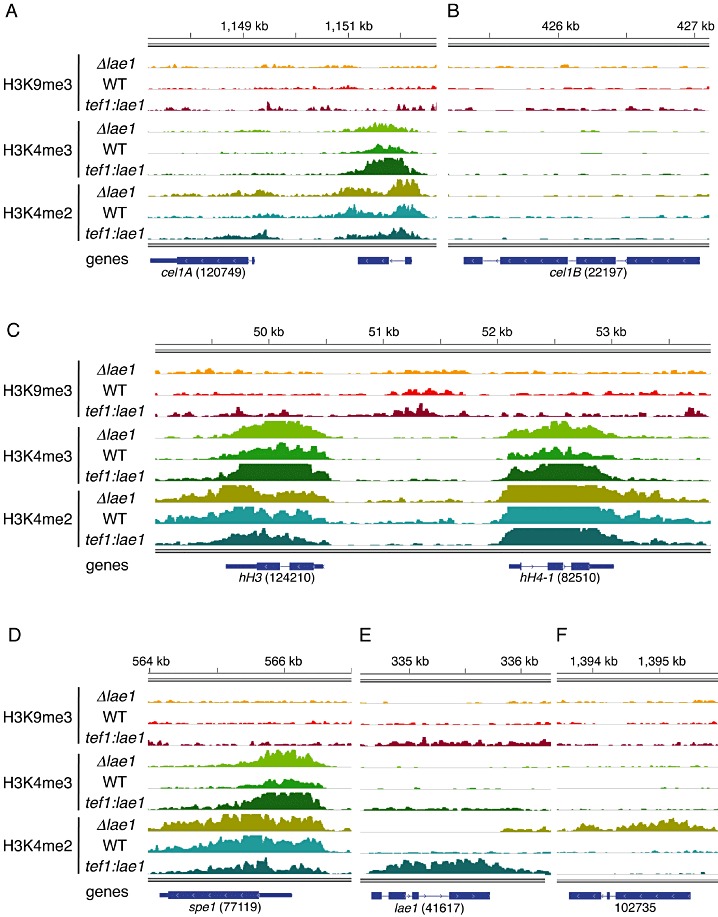
Selected results of ChIP-seq with antibodies against H3K9me3, H3K4me3 and H3K4me2 in Δ*lae1* (CPK3793), wild type (QM 9414) and the LAE1-overexpressing *tef1:lae1* strain CPK4086. Genes of interest are shown below regions of enrichment (*y*-axis scale, 0–30 for all tracks, all data were normalized for relative abundance). A. H3K4me3 is mildly enriched in the 5′ region of *cel1a* but not nearly as strongly as in the neighbouring gene, which is not affected by changes in LAE1 expression. B. The most common pattern of CAZyme gene histone modifications tested here was no significant enrichment under any condition, represented here by *cel1b*. C. In contrast, highly expressed genes, like *hH3* and *hH4-1* show both H3K4me2 and -me3 but no H3K9me3 enrichment in a *lae-1-*independent manner. D. The same is true for a typical metabolic gene, *spe1*, the gene for ornithine decarboxylase. E. The *lae1* gene serves as control, as no signal is detected in Δ*lae1*, but H3K4me2 is enriched in the *tef1:lae1* strain. F. A predicted gene encoding a protein with a carbohydrate-binding motif (protein ID 102735) shows enrichment of H3K4me2 only in the Δ*lae1* strain.

ChIP with H3K4me2, H3K4me3 and H3K9me3 antibodies resulted in the expected patterns in most transcribed genes for all three strains examined in this study, i.e. H3K4me2 and H3K4me3 enrichment and absence of H3K9me3 (a detailed, genome-wide analysis of the results is in preparation). However, only one CAZyme gene (*cel5B*) showed enrichment of H3K4 methylation in wild type and *tef1:lae1*, with concomitant reduction in Δ*lae1*, and one gene (*cel1A*) showed some enrichment of H3K4me2 in all strains without enrichment of H3K4me3 (Fig. 10A). There was no clear change in histone modifications within any of the CAZyme genes that we showed by microarray analysis to have expression levels that are strongly affected by either the absence or overexpression of LAE1. As expected, several CAZyme genes showed minor enrichment of H3K4 methylation in the *tef1:lae1* overexpression strain (*xyn2*, *cip1*, *cel6A*, *cel7B*, *cel61B*; data not shown). However, the most commonly observed pattern for these genes (*cel12A*, *cel61A*, *cel45A*, *cel3D*, *cel3C*, *cel5A*, *cel7A*, 108671/GH3 β-glycosidase, 111874/swollenin-like, *cbm13*, 123992/swollenin, *xyn1*, *xyn3*, *cip2*; see *cel1B*, Fig. 10B) was no enrichment with any of the histone marks we tested. Also, no enrichment was seen at the *xyr1* locus (data not shown). ChIP-seq results were validated by region-specific PCR of most regions and results were similar, i.e. no enrichment differences for any of the histone marks in the three different strains in any of the regions tested (data not shown).

The ChIP-seq was successful, as most genes showed the expected patterns for H3K4 and H3K9 methylation (e.g. see the genes for histone H3 and H4-1, and ornithine decarboxylase; Fig. 10C and D). Also, the *lae1* gene served as a convenient control as in Δ*lae1* there was no enrichment of any kind observed (the flat line indicates absence of the segment from the genome; Fig. 10E), while in *tef1:lae1* both H3K4me2 and H3K4me3 were enriched at *lae1*, which is consistent with overexpression of the gene. Lastly, one gene for a putative carbohydrate-binding protein (protein ID 102735) was more highly enriched for H3K4me3 in Δ*lae1* but not in any other condition. Taken together, this analysis suggests that LAE1 does not directly affect the balance of H3K4 and H3K9 methylation at the CAZyme genes in *T. reesei*.

### LAE1 is essential for *T. reesei* asexual sporulation

**Figure 11:**
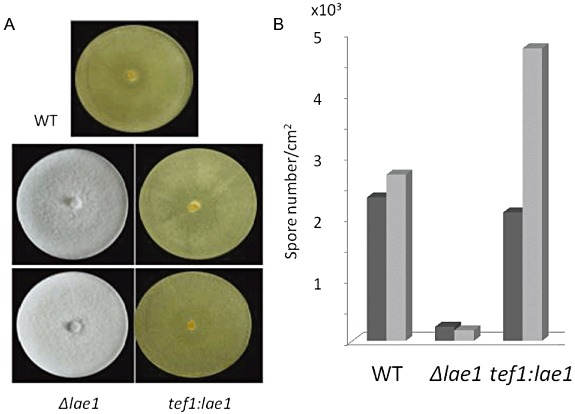
LAE1 affects sporulation in *T. reesei*. (A) Phenotype of *T. reesei* QM 9414 (WT), Δ*lae1* (CPK3793 on top, and CPK4086 below) and *lae1*OE strains (CPK4086) on top, CPK3791 below); (B) effect of light on sporulation in *T. reesei* WT, Δ*lae1* (CPK3793) and *lae1*OE (CPK4086) strains. Only one strain is shown, but consistent data have been obtained with at least two more strains of both *lae1* genotypes.

Manipulation of *lae1* gene expression in *T. reesei* caused striking phenotypic changes: delta-*lae1* strains can readily be observed on plates by the lack of the characteristic yellow pigment that is produced by *T. reesei*, and by the significantly reduced sporulation (Fig. 1). Overexpression of *lae1* (OE*lae1*) recovered the pigmentation, and led to even increased sporulation (Fig. 11A). A quantitative analysis showed that sporulation was reduced to 5% of the control in delta-lae1, and doubled in the OElae1 strain compared with the control (Fig. 11B). Interestingly, sporulation in the wild type and the delta-*lae1* strain was not effected by light or darkness, but the increased sporulation in the OElae1 strain was only apparent in light.

We consequently wondered whether this difference would also be reflected in a similarity between the conidiating and *lae1*-effected transcriptome. Metz *et al*. (2011) have recently reported that 900 genes are significantly expressed in *T. reesei* during conidiation. When they were compared with the transcriptome of the delta-*lae1* strain, 254 genes (i.e. 47.9% of all genes upregulated during conidiation and 33.2% of the genes found to be downregulated in delta-*lae1*) correlated between the two conditions (i.e. upregulated during sporulation and downregulated in the delta-*lae1* strain; and vice versa) (Table S2).

## Discussion

Data presented here show that the putative protein methyltransferase LAE1 influences cellulase gene transcription in *T. reesei*, and may thus represent a novel approach for cellulase overproduction and strain improvement by recombinant techniques in this fungus. Although the use of LaeA for increasing secondary metabolite production by fungi has been proposed (Keller and Bok, 2006; Keller *et al*., 2007), we are not aware of any successful demonstration of this principle in an industrially relevant fungal species. Since we have used one of the ‘early’ strains from the *T. reesei* mutant pedigree, *T. reesei* QM 9414 (Le Crom *et al*., 2009), we cannot be certain that high-cellulase producing strains exhibit the same degree of increase in cellulase formation as observed here with strains that constitutively expressed *lae1*. However, we note that genome-wide analysis of the currently highest cellulase producing strain in the public domain –*T. reesei* RUT C30 – did not reveal mutations in genes related to chromatin-level gene regulation, and its *lae1* locus was intact (Le Crom *et al*., 2009). Our data suggest an attractive new approach for increasing total cellulase activity in *T. reesei* in a single step. The only other means to globally increase production of all cellulases – by increasing the expression or activity of the cellulase- and hemicellulase-specific transcriptional regulator XYR1 (Stricker *et al*., 2006) – has so far not yielded significant improvements (Mach-Aigner *et al*., 2008). Interestingly, *xyr1* itself is located in one of the CAZyme clusters, and downregulated in the Δ*lae1* strain during growth on lactose (Table S1). Data from this article further showed that overexpression of either *xyr1* or *lae1* under constitutive promoters cannot rescue the impairment of cellulase gene expression by a deletion in the other gene respectively. This suggests that LAE1 is involved in control of transcription of the cellulase genes and that of their common regulator. The signalling pathway by which the presence of any inducer of cellulase activity is communicated to the *T. reesei* transcriptional machinery is not known yet. Its identification will shed light on how LAE1 functions in the activation of transcription of cellulase-encoding genes in this fungus.

While the effect of LAE1 on *T. reesei* CAZyme gene expression has clearly been demonstrated in this article, one hypothesis was falsified, i.e. that genomic clusters of cellulase genes (Martinez *et al*., 2008) would serve as targets for LAE1-mediated counteraction of H3K9 methylation, and thus binding of HP1 and generation of heterochromatin. In fact, the LAE1-independent absence of H3K9me3 from the CAZyme loci, and the very slight enrichment of H3K4 methylation in LAE1 overproducing *T. reesei* mutants suggests that the cellulase genes are only little affected by repressive and non-repressive chromatin, and thus LAE1 activates CAZyme gene transcription by a mechanism independent of histone H3K4 and H3K9 methylation. Unfortunately, the target protein of methylation by LAE1 has not yet been identified in any organism (Bayram and Braus, 2012). We must also note that – although ChIP analysis of heterochromatic marks in *A. nidulans laeA*Δ strains had revealed a dramatic increase in H3K9me3 and HepA binding at secondary metabolism clusters (Reyes-Dominguez *et al*., 2010) – a direct effect of LaeA on histone modification still awaits biochemical evidence.

While the precise mechanism of action of LAE1 remains to be identified, we speculate that it could be related to the linkage between asexual sporulation and CAZyme gene transcription in *T. reesei* (Metz *et al*., 2011): as we have shown in this article, one of the most striking phenotypes of *lae1*-mutants in *T. reesei* is the almost complete absence of sporulation in the Δ*lae1* and hypersporulation in *tef1:lae1* strains, a phenomenon already observed in some other fungi (Bok *et al*., 2005; Kosalková*et al*., 2009). The fact that approximately half of the genes upregulated during asexual sporulation are identical to a third of the genes downregulated in delta-*lae1* renders asexual development a major target in *T. reesei*. Asexual sporulation triggers massive CAZyme gene expression in *T. reesei* in an inducer-independent but XYR1-dependent way (Metz *et al*., 2011), and sporulation is commonly observed at later stages of cellulase formation on lactose which was absent from the Δ*lae1* strain and increased in the *tef1:lae1* mutant in this study (data not shown). Absence of conidial cellulases renders them unable to germinate on cellulose as a carbon source (Metz *et al*., 2011). We consider it therefore possible that the regulation of sporulation is the prime target of LAE1, and that the effect on cellulase gene expression occurs by a signal for CAZy gene expression created during sporulation. We should note that sporulation is also commonly observed during submerged growth of *Trichoderma* spp. on carbon sources inducing cellulase formation such as cellulose or lactose (Lewis and Papavizas, 1983).

Independent of the underlying mechanism, our results demonstrate the obvious advantage of genomic clustering of the *T. reesei* cellulases, although the evolutionary mechanisms that have led to this situation still remain unclear. In the case of secondary metabolite biosynthetic genes, clustering has been suggested to reflect their evolutionary history (Zhang *et al*., 2004; Keller *et al*., 2005). One model, the ‘selfish cluster’ hypothesis, requires that selection occurs by promoting the maintenance of the cluster as a unit, e.g. by horizontal transfer events (Walton, 2000). As far as the major cellulases and hemicellulases of *T. reesei* are concerned; however, there is no evidence or indication for horizontal gene transfer. Alternatively, it has been suggested that clusters are maintained by the operation of co-regulatory mechanisms. This is essentially what is observed with cellulases and hemicellulases, which are co-induced by similar signals (Foreman *et al*., 2003) and controlled by a single major transcriptional regulator XYR1 (Stricker *et al*., 2006).

In summary this article describes the successful attempt to increase production of all *T. reesei* cellulases by modulation of a single gene. While reaching a cost-efficient application of second-generation biofuel production still depends also on an improvement of several of the steps involved, e.g. biomass pre-treatment, enzyme composition and pentose fermentation (Margeot *et al*., 2009; Xu *et al*., 2009), our data suggest a means to solve one of the essential steps, i.e. the need for increasing enzyme production. Learning how LAE1 becomes active and identifying its actual target protein in *T. reesei* will contribute additional or alternative tools for improvement of cellulase production at the chromatin level.

## Experimental procedures

### Strains used in this work and their cultivation

*Trichoderma reesei* QM 9414 (ATCC 26921), an early cellulase producing mutant and *T. reesei* KU70 (Guangtao *et al*., 2009), a derivative of the QM 9414 uridine auxotrophic *pyr4*-negative strain TU-6 (ATCC MYA-256) (Gruber *et al*., 1990) which bears a deletion in the *tku70* gene and is thus deficient in non-homologous end joining, were used throughout this work. *Escherichia coli* JM109 (Promega, Madison, Wisconsin) was used for plasmid construction and amplification.

For cellulase production, *T. reesei* was grown in Mandels-Andreotti medium (Akel *et al*., 2009) using Avicel cellulose, lactose, oat spelts xylan or glycerol as a carbon source (1%, w/v) as stated at the respective results. Induction of cellulases by sophorose (0.5 mM) in pre-grown, washed mycelia was performed as described (Sternberg and Mandels, 1979).

### Phylogenetic identification of *T. reesei* LAE1

For the identification of the *T. reesei* LaeA orthologue the LaeA proteins from *A. nidulans* and *A. fumigatus* were used to retrieve the respective orthologues from all other *Aspergilli* by blastp. These proteins were then collectively used to retrieve the *C. immitis* LaeA. The latter was used to identify the corresponding orthologues from the three *Dothidiomycete* taxa *Phaeospora nodorum, Pyrenophora tritici repentis* and *Cochliobolus heterostrophus*. Using the latter, we identified the proteins from the three *Neurospora* spp. followed by the identification of the LaeA orthologues in *Chaetomium globosum* and *Fusarium* spp. before we finally identified the LaeA orthologues in the three *Trichoderma* spp. The correctness of the best hits was always cross-checked by reversed blastp and these best hits always exhibited a reliable aa-identity of > 70%. For gene/protein sequences used in this approach, see Table S3.

### Nucleic acid isolation and hybridization

Fungal mycelia were harvested by filtration, washed with distilled cold water, frozen and ground under liquid nitrogen. For extraction of genomic DNA, plasmid DNA and RNA, purification kits (Wizard Genomic DNA Purification Kit, PureYield Plasmid Midiprep System and SV Total RNA Isolation System, respectively, all from Promega) were used according to the manufacturer's protocol. cDNA synthesis from the predicted *lae1* mRNA was done with a RevertAid^TM^ H Minus First strand cDNA Synthesis Kit (Fermentas, MA), using the primers Lae1ATG and LAE1stop (Table S4). Standard methods were used for electrophoresis, blotting and hybridization of nucleic acids.

### Construction of *T. reesei* strains with altered *lae1* alleles

To study the function of LAE1, we constructed *T. reesei* strains in which *lae1* was deleted and strains which expressed *lae1* under the strong constitutive expression signals of the *tef1* (translation elongation factor 1-alpha encoding) promoter region (Akel *et al*., 2009).

To delete the *lae1* gene of *T. reesei,* the 1.2 kb *lae1* coding region was replaced by the *T. reesei pyr4* (orotidine 5′-phosphate decarboxylase-encoding) gene. This was performed by amplifying around 1 kb of the up- and downstream non-coding region of *lae1* from genomic DNA of *T. reesei* QM9414 using the primer pairs given in Table S4. The two resulting PCR fragments were digested with HindIII/XhoI (upstream region) and ApaI/XhoI (downstream region) and ligated into an ApaI/HindIII restricted vector pBluescript SK(+) (Stratagene, La Jolla, California), followed by the insertion of the 2.7 kb SalI fragment of *T. reesei pyr4* in the XhoI site resulting in pRKA_D41617pyr4.

For expression of *lae1* under a strong constitutive promoter in *T. reesei* QM 9414 and in the *T. reesei Δxyr1* strain (Stricker *et al*., 2006), we amplified a 1820 bp *lae1* PCR fragment including the coding and terminator region with the oligonucleotides TrLae1ATGCla and TrLae1TermHind (Table S4) and inserted the fragment downstream of the *tef1* promoter region (GenBank Accession No. Z23012.1) into the ClaI/HindIII sites of pLH1hphtef1 resulting in vector pRKA_OE41617hph*,* which contains the *E. coli* hygromycin B phosphotransferase (*hph*) under *T. reesei* expression signals as selection marker (Akel *et al*., 2009).

To construct a strain constitutively expressing *xyr1* in a delta-*lae1* background, the plasmid pRLMex30 (Mach *et al*., 1994) was digested with XbaI and HindIII, removing the *hph* coding region and *cbh2* terminator region (2066 bp) and leaving the *pki1* promoter. Next a 2.4 kb fragment containing about 800 bp of the *trpC* promoter, the *nptII* coding region and about 700 bp of the *trpC* terminator was amplified from pII99 (Namiki *et al*., 2001) using the primers GenFW (CCTCTTAACCTCTAGACGGCTTTGATTTCCTTCAGG) and GenRV (TGATTACGCCAAGCTTGGATTACCTCTAAACAAGTGTACCTGTG). The two fragments were joined by In-Fusion recombination (Clontech) resulting in the plasmid pPki-Gen, which was verified by digestion with SacII and XbaI+HindIII. Then the *xyr1* coding and terminator region was amplified using the primers XyrFW (CCTCTTAACCTCTAGAATGTTGTCCAATCCTCTCCGTCG) and XyrRV (ATCAAAGCCGTCTAGATCTACAGCCATGCTCATCGTGC). The resulting 3.5 kb fragment was inserted into the XbaI site of pPki-Gen by In-Fusion recombination (Clontech) yielding plasmid pPki-Xyr, which was verified by digestion with XbaI and sequencing. This plasmid was then used to transform the delta-*lae1* strain of *T. reesei*.

### Fungal transformation

All vectors constructed were verified by sequencing. The strains were purified twice for mitotic stability, and integration of the expression cassettes was verified by PCR analysis. Protoplast preparation and DNA mediated transformation was described (Guangtao *et al*., 2009).

Gene copy numbers of the integrated constructs were determined by Southern analysis, using chromosomal DNA cleaved with BamHI. This enzyme cleaves within *lae1*, and at the native locus gives rise to two fragments (2.3 and 4 kb respectively; using the *lae1* cDNA as a probe). No additional BamHI sites occur in the *tef1:lae1* gene construct. Thus, in case of an intact wild-type locus, integrated *tef1:lae1* copies are visible by additional pairs of *lae1* restriction fragments.

### Biochemical assays

Cellulase enzyme activities were determined using carboxymethylcellulose (1%, w/v) as described (Vaheri *et al*., 1979). Protein concentrations in the culture supernatant were determined by the method of Bradford (1976).

### Transcriptome analysis of *lae1* loss of function and *lae1* overexpression

Mycelia were ground in liquid nitrogen using a mortar and pestle. Total RNAs were extracted using TRIzol® reagent (Invitrogen Life Technologies, Carlsbad, CA, USA), according to the manufacturer's instructions, and then purified using the RNeasy MinElute Cleanup Kit (Qiagen, Hilden, Germany). The RNA quality and quantity were determined using a Nanodrop spectrophotometer. High-quality purified RNAs were submitted to Roche-NimbleGen (40 µg per three-microarray set) where cDNAs were synthesized, amplified and labelled and then used for subsequent hybridization.

A high density oligonucleotide (HDO) microarray (Roche-NimbleGen, Madison, WI, USA) was constructed, using 60-mer probes representing the 9.129 genes of *T. reesei*. Microarray scanning, data acquisition and identification of probe sets showing a significant difference (*P* = 0.05) in expression level between the different conditions were performed by Roche-NimbleGen (http://www.nimblegen.com). Values were normalized by quantile normalization (Bolstad *et al*., 2003) and the RMA algorithm (Irizarry *et al*., 2003); this was done by Nimblegen. After elimination of transcripts that exhibited an SD > 20% within replicates, the FDR (Benjamini Hochberg) method (Benjamini and Hochberg, 1995) was used to assess the significance of values. Transcripts showing significantly downregulated expression in the Δ*lae1* strain (at least twofold changes) were annotated manually. The data set was also manually screened for the downregulation of genes encoding carbohydrate active enzymes to at least twofold changes. Gene accession numbers were annotated according to version 2 of the *T. reesei* genome assembly (http://genome.jgi-psf.org/Trire2/Trire2.home.html), and ambiguous cases annotated manually.

The microarray data and the related protocols are available at the GEO website (http://www.ncbi.nlm.nih.gov/geo/) under Accession No. GSE22687 (platform GPL10642).

### Analysis of genomic clustering of transcripts

*Trichoderma reesei* genes have not yet been mapped to chromosomes, but their appearance on genomic scaffolds is known. In order to identify whether the significantly regulated transcripts would be clustered to particular areas on these scaffolds, we aligned them onto an ordered list of genes on the individual scaffolds. Distances (= numbers of genes) between positive hits were recorded. Clustering of transcripts was considered to appear if the distance between them was at least threefold smaller than the average distribution of the 769 significantly regulated transcripts among all genes (9143), i.e. a third of 11.9 = 3.9.

### Real-time PCR

DNase treated (DNase I, RNase free; Fermentas) RNA (5 µg) was reverse transcribed with the RevertAid™ First Strand cDNA Kit (Fermentas) according to the manufacturer's protocol with a combination of oligo-dT and random hexamer primers (Table S5). All real-time RT-PCR experiments were performed on a Bio-Rad (Hercules, CA) iCycler IQ. For the reaction the IQ SYBR Green Supermix (Bio-Rad, Hercules, CA) was prepared for 25 µl assays with standard MgCl_2_ concentration (3 mM) and a final primer concentration of 100 nM each. All assays were carried out in 96-well plates. Determination of the PCR efficiency was performed using triplicate reactions from a dilution series of cDNA (1; 0.1; 0.01; 0.001). Amplification efficiency was then calculated from the given slopes in the IQ5 Optical system Software v2.0. Expression ratios were calculated using REST© Software (Pfaffl *et al*., 2002). All samples were analysed in at least two independent experiments with three replicates in each run.

### Chromatin immunoprecipitation (ChIP) and ChIP sequencing

To carry out ChIP sequencing with *T. reesei* we adapted a protocol developed for *Neurospora crassa* (Tamaru *et al*., 2003; Smith *et al*., 2011). QM 9414, Δ*lae1* and *tef1::lae1* strains were grown for 5 days in the dark on 2% PDA medium and spores then harvested. Flasks with 50 ml of lactose medium were inoculated with either 1 × 10^5^ or 1 × 10^6^ spores ml^−1^ and grown in the dark for 26 h. All further steps were as described previously (Tamaru *et al*., 2003). DNA obtained by ChIP was suspended in 30 µl and used either for region-specific ChIP with primers for CAZyme genes (sequences available upon request) or for the construction of ChIP-seq libraries (Pomraning *et al*., 2009). We obtained 1.4–4.8 million mapped reads (between 76% and 98% of the total reads) for the nine libraries we sequenced (three strains × three antibodies). The antibodies used were from Active Motif (H3K4me3, 39159; H3K9me3, 39161) and Upstate/Millipore (H3K4me2, 07-030). We used one additional H3K9me3 antibody from abcam (ab8898), which resulted in less enrichment than with the Active Motif antibody. Genome-wide analysis of the ChIP-seq results is in preparation.

### Statistical analysis

Basic statistical methods such as multiple regression analysis and analysis of variance (anova) as well as multivariate exploratory techniques (cluster and factor analyses) were performed using Statistica 6.1 (StatSoft, Tulsa, OK, USA) data analysis software system.
