# Supplementary material for: The putative protein methyltransferase LAE1 controls cellulase gene expression in Trichoderma reesei
Source: Mol Microbiol. 2012 Jun;84(6):1150–64. doi: 10.1111/j.1365-2958.2012.08083.x (PMC3370264; doi:10.1111/j.1365-2958.2012.08083.x)
Supplement: Supplementary file 1 [file mmi0084-1150-SD1.pdf]

Supplementary Table S1: CAZome genes that are upregulated in the *tef1:lae1* strain

protein ID	Identification	QM 9414 [log_2]	S.D. [log2]	*tef1:lae1* [log2]	S.D. [log2]	*tef1:lae1* (-fold)	p-value
3094	GH30 ß-1,6-glucanase	5.59588	0.12234	7.17447	0.6662	2.986 up	0.0223
5836	GH2 ß-mannosidase	10.7385	0.03373	11.79068	0.42011	2.073 up	0.0321
22129	GH61 celluilose monooxygenase CEL61B	10.91782	0.01727	12.59391	0.67915	3.195 up	0.041
27219	GH27 alpha-galactosidase	3.81507	0.46426	5.24526	0.10711	2.694 up	0.00533
41768	GH16 glycoside hydrolase	6.04602	0.26971	7.87103	0.65437	3.543 up	0.025
46816	GH3 ß-glucosidase	8.70765	0.07007	10.40969	0.23061	3.253 up	0.000655
47268	GH3 ß-glucosidase.	11.20929	0.0589	12.75641	0.28754	2.922 up	0.00411
49193	GH17 ß-glycosidase	12.94412	0.03577	13.9912	0.33158	2.066 up	0.0214
49976	GH45 endoglucanase CEL45=EGL5	8.40415	0.20013	13.33607	0.38552	30.525 up	0.000107
50215	GH16 ß-glycosidase, one transmembrane domain	11.3347	0.1033	12.58219	0.2964	2.374 up	0.000381
55802	GH76 alpha-1,6-mannanase	8.68349	0.01567	11.45617	0.65402	6.833 up	0.00589
55999	GH27 α-galactosidase	8.14024	0.03952	9.36371	0.24875	2.335 up	0.000868
56448	GH18 endochitinase CHI18-11	6.8206	0.09009	8.23413	0.10536	2.663 up	0.0000997
56894	GH18 endochitinase CHI18-10	2.87534	0.22714	4.25771	0.10343	2.606 up	0.00122
57179	GH88 glycosyl hydrolase	5.09815	0.27384	8.17708	0.56865	8.449 up	0.00268
58450	GH3 ß-xylosidase.	4.41688	0.14502	5.64895	0.31322	2.349 up	0.0129
58887	GH78 alpha-L-rhamnosidases	3.37525	0.1207	4.86028	0.11271	2.799 up	0.00046
59791	GH18 endochitinase CHI18-15	2.71721	0.04635	5.03302	0.41795	4.978 up	0.00233
60635	GH92 α-1,2-mannosidase	5.36203	0.26278	10.04624	1.90555	25.709 up	0.0186
65162	GH18 endochitinase CHI18-1b	10.93982	0.18638	13.36774	0.14046	5.381 up	0.000338
65380	1, 2-alpha-mannosidase	10.51249	0.12254	11.92898	0.25541	2.669 up	0.00339
69276	GH30 ß-glycoside hydrolase	7.51479	0.17067	11.80902	0.71743	19.619 up	0.000992
70186	GH28 polygalacturonase	5.05832	0.23144	6.71626	0.48761	3.155 up	0.00759
70845	GH55 ß-1,3-glucanase	9.03833	0.11074	11.58816	0.16389	5.855 up	0.00032
71245	GH18 endochitinase CHI18-1a	4.31858	0.19861	5.50133	0.43093	2.270 up	0.0356
71554	GH5 ß-glycosidase	3.70787	0.25126	5.34567	0.12291	3.111 up	0.000665
72071	CBM13 Carbohydrate-Binding Module Family 13	5.70232	0.3653	7.48403	0.61624	3.438 up	0.0249

72567	GH6 cellobiohydrolase CEL6A=CBH2	12.99037	0.15137	15.25272	0.15465	4.797 up	0.0000595
73005	GH79 ß-glycosidases	2.6602	0.01767	4.49246	0.09096	3.560 up	0.0000908
73101	GH16 ß-glycosidase	4.8095	0.11944	6.29322	0.1155	2.796 up	0.000564
73179	GH95 α-fucosidase	6.98958	0.06552	8.78224	0.42735	3.464 up	0.00832
73632	CE5 acetylxylan esterase AXE1	7.18943	0.14111	11.0991	0.28218	15.028 up	0.0000973
73638	CIP1	9.0132	0.17398	13.63062	0.37794	24.546 up	0.000107
73643	GH61 cellulose monooxygenase CEL61A	7.80786	0.10156	12.32581	0.15622	22.910 up	0.0000299
76210	GH62, abf2, a-L-arabinofuranosidase	7.01599	0.1865	10.47392	1.48912	10.988 up	0.00526
76672	ß-glucosidase 1 (EC 3.2.1.21)	7.04683	0.1593	11.84275	1.68676	27.778 up	0.0151
77299	GH2 exo-ß-D-glucosaminidase	8.93389	0.19956	10.1115	0.50636	2.262 up	0.00312
80340	GT32 α-mannosyltransferase	9.93872	0.1123	12.59314	0.15565	6.295 up	0.000242
81087	GH5 glycoside hydrolase	10.24147	0.0941	11.84336	0.55476	3.035 up	0.000925
82235	GH31 α-glucosidase B	12.75967	0.10122	14.26778	0.17921	2.844 up	0.00103
103049	GH28 polygalacturonase	5.31208	0.23593	6.61591	0.24359	2.468 up	0.00177
105448	CBM13 carbohydrate-binding protein	3.29043	0.21797	5.79799	0.39703	5.686 up	0.00229
108346	GH18 endochitinase CHI18-8	3.45563	0.07839	5.28355	0.25648	3.550 up	0.00141
108776	GH55 ß-1,3-glucanase	5.42228	0.13961	6.7659	0.23256	2.537 up	0.00257
109278	GH24 related to phage lysozymes	7.0068	0.14823	9.85215	0.82357	7.186 up	0.0163
110259	PL7 alginate lyase like protein	3.42471	0.11824	4.77698	0.11552	2.553 up	0.000728
110317	GH18 endochitinase CH18-17	3.11463	0.09064	4.81687	0.11685	3.254 up	0.000242
110848	protein with GH23 modules, glycoside hydrolase not yet assigned to a family	3.31933	0.16332	5.57066	0.5235	4.761 up	0.00532
110894	GH5 ß-1,4-endogalactanase	7.00883	0.12059	9.29206	0.9065	4.867 up	0.0145
111733	GH92 alpha-1,2-mannosidase	3.39345	0.32359	4.95686	0.10151	2.955 up	0.00107
111849	GH30 endo-ß-1,4-xylanase XYN4	9.07927	0.02793	10.11854	0.07768	2.055 up	0.000186
112140	GH28 polygalacturonase	4.07985	0.32754	8.3271	0.38039	18.991 up	0.00042
112392	GH11 candidate xylanase (XYL5)	4.89618	0.07513	6.92671	0.36806	4.085 up	0.00259
120229	GH10 xylanase XYN3	7.1759	0.11749	13.2519	0.8036	67.461 up	0.000643
120312	GH5 endoglucanase Cel5A	10.85671	0.20952	14.80739	0.26443	15.462 up	0.0000716
120749	GH1 CEL1A ß-glucosidase	13.19302	0.01401	14.50099	0.0573	2.475 up	0.000048

120873	GH71 α-1,3-glucanase, 2 CBM	4.82384	0.29231	8.28327	0.26109	11.000 up	0.000374
120961	GH61 endoglucanase CEL61B	8.6541	0.09645	13.06312	0.69301	21.244 up	0.000327
121127	GH3 BXL1 ß-xylosidase	10.11473	0.01254	12.3645	0.46332	4.756 up	0.000377
121746	GH55 exo-ß-1,3-glucosidase GLUC78	10.82838	0.07343	12.79041	0.48476	3.896 up	0.0105
122081	GH7 endoglucanase CEL7B=EGL1	8.68794	0.2276	13.75508	0.40052	33.524 up	0.000204
122780	GH28 polygalacturonase	11.30576	0.03609	13.91132	0.27322	6.086 up	0.000798
123232	GH12 endoglucanase Cel12A	10.75466	0.19955	14.31326	0.26129	11.782 up	0.0000743
123639	GH64 ß-1,3-glucanase	3.67615	0.23266	5.06862	0.24667	2.625 up	0.00331
123818	GH11 xylanase XYN2	11.69446	0.06929	13.01123	0.60738	2.491 up	0.000747
123940	methyl-glucuronoyl esterase CIP2	4.83643	0.37262	10.17903	1.68645	40.577 up	0.00715
123989	GH7, cellobiohydrolase Cel7A=CBH1	14.27507	0.04409	15.58843	0.08791	2.485 up	0.0000494
123992	swollenin	11.22247	0.1043	13.13211	0.65201	3.757 up	0.00312
124043	GH18 endochitinase CHI18-14	3.51068	0.38967	6.63093	0.22616	8.695 up	0.000612

The table shows the expression levels (in $\log_2$) of the lae1OE strain CPK4086 and QM 9414. “fold” indicates the enhancement in strain CPK4086;

Supplementary Table S2. Genes that are >2-fold downregulated in delta-*lae1* and >2-fold-upregulated during conidiation*

Protein ID	***Annotation***
70800	elastinolytic metalloproteinase
66551	AAA ATPase
46128	AAA+-type ATPase
69956	Alcohol dehydrogenase, class V
80659	alcohol oxidase AOX1
57940	alternative oxidase aox1
5970	Amidase
69863	Amidases
67806	Amino acid transporter
64971	Amino acid transporters
81420	Argonaute siRNA chaperone (ARC) complex subunit
70803	bifunctional catalase/peroxidase
59151	BYS1 domain protein
119826	C2H2 and homeobox transcription factor
112538	C2H2 transcription factor
111567	C2H2 transcription factor
120597	C2H2 transcriptional regulator (Egr2 ?)
4171	Ca2+ transporter
2316	caleosin related protein
64370	calpain-like protease
67538	Catalase
120371	Catalase
54219	CE acetyl xylan esterase
73632	CE acetyl xylan esterase AXE1
123659	cell wall protein. Distantly related to S. cerevisiae Pir3p.
73638	CIP1
67964	Cytochrome P450 CYP2 subfamily

64900	Cytochrome P450 CYP3/CYP5/CYP6/CYP9 subfamilies
59377	Cytochrome P450 CYP3/CYP5/CYP6/CYP9 subfamilies
66534	Cytochrome P450 CYP3/CYP5/CYP6/CYP9 subfamilies
75713	Cytochrome P450 CYP4/CYP19/CYP26 subfamilies
38749	Cytochrome P450 CYP4/CYP19/CYP26 subfamilies
52489	cytosin/purin permease
123946	dehydrogenase associated with cellulase signal transduction (PMID: 15288024)
70961	dienelactone hydrolase
65883	D-isomer-specific 2-hydroxy acid dehydrogenase
61553	D-xylulose 5-phosphate/D-fructose 6-phosphate phosphoketolase
82662	Epl1
61078	esterase/ lipase
106697	esterase/lipase, HGT
105823	FAD binding domain protein
66726	FAD binding protein
4114	FAD linked oxidase
104211	FKBP-type peptidyl-prolyl cis-trans isomerase
111716	Flavonol reductase/cinnamoyl-CoA reductase
111881	flavoprotein monooxygenases
72922	GABA permease, 12 TM
104222	GCN5-related acetyltransferase
112665	GCN5-related N-acetyltransferase
121418	G-D-S-L lipase/acylase
56840	GFO_IDH_MocA dehydrogenase
120749	GH1 CEL1A ß-glucosidase
120229	GH10 xylanase XYN3
123818	GH11 xylanase XYN2
123940	GH115 glucuronoyl esterase CIP2
123232	GH12 endoglucanase Cel12A
65333	GH15 candidate a-glycosidase (trehalase ?)

69245	GH2 ß-mannosidase
72632	GH27-α-galactosidase
103049	GH28: candidate polygalacturonase
121127	GH3 ß-xylosidase BXL1
69276	GH30 glucuronoxylan hydrolase
69944	GH31 a-glucosidase
4561	GH32, candidate a-glycosyltransferase
123226	GH37 trehalase
120312	GH5 endoglucanase Cel5A
81087	GH5 glycoside hydrolase
72567	GH6 cellobiohydrolase CEL6A
73643	GH61 cellulase activating protein CEL61A
120961	GH61 cellulase activating protein CEL61B
76210	GH62, abf2, a-L-arabinofuranosidase
72526	GH67 α-glucuronidase
122081	GH7 CEL7B endoglucanase 1
123989	GH7 cellobiohydrolase Cel7A
49081	GH74 xyloglucan hydrolase
71394	GH79 related to b-glycosidases
74198	GH92 α-1,2-mannosidase
5807	GH95 α-fucosidase
112022	glutathione-S-transferase
44366	Glycosylphosphatidylinositol-specific phospholipase C
72259	GPCR, RgsA-type
77547	GT1 glycosyltransferase
63756	helicase, DEAD-box superfamily
103189	HET protein
106171	HET protein
80142	HSP104 and related ATP-dependent Clp proteases
122363	Hsp26/Hsp42

46285	Hsp26/Hsp42
62100	Hsp30
55362	HSP70/HSC70, HSP70 superfamily
54352	hypothetical protein
123468	IMP dehydrogenase
76034	Iron/ascorbate family oxidoreductases
105968	Iron/ascorbate family oxidoreductases
105342	Iron/ascorbate family oxidoreductases
5182	iron-dependent peroxidase
77423	Jacalin-like lectin
105518	K+ channel
122820	Kynurenine aminotransferase, glutamine transaminase K
44175	lactate pyruvate transporter
55240	large-conductance mechanosensitive channel
104599	Mandelate racemase/muconate lactonizing enzyme
74194	mannitol dehydrogenase LXR1
58701	Mannose-6-phosphate isomerase
75165	MSF transporter
54972	MSF transpoorter
44956	MSF transporter
54036	MSF transporter
69957	MSF transporter
70933	MSF transporter
78585	MSF transporter
3405	MSF transporter
26642	MSF transporter
57749	MSF transporter
62380	MSF transporter (galactose permease?)
54239	Multicopper oxidases
44476	Multidrug resistance-associated protein, ABC superfamily

67971	MYND-type Zn-finger protein
76366	NADH:flavin oxidoreductase/12-oxophytodienoate reductase
60352	NADH-dehydrogenase (ubiquinone)
53079	NADP/FAD dependent oxidoreductase
109538	nZIP (ATF2 ?)
124097	phenazine biosynthesis PhzC/PhzF-type epimerase
124056	phosphatidic acid phosphatase
64959	phosphatidyl synthase
121498	Phosphatidylserine decarboxylase
123572	Phospholipase A2
67579	phospholipase A2
122824	PHT11-type GCPRs
73618	PKS
73621	PKS
82327	Pleiotropic drug resistance proteins (PDR1-15), ABC superfamily
82105	Pleiotropic drug resistance proteins (PDR1-15), ABC superfamily
111245	polysaccharide lyase; distantly related to chondroitin lyases
58418	Predicted unusual protein kinase
5647	PTH11-type GPCRs
47315	P-type ATPase
74282	QI74 orthologue
111053	retrograde regulation protein 2
72137	sceleton binding protein
59364	Sexual differentiation process protein ISP4
106164	short chain dehydrogenase/reductase
77202	short chain dehydrogenase/reductase
120911	short chain dehydrogenase/reductase
3055	short chain dehydrogenase/reductase
23090	short chain dehydrognease/reductase
34297	Stress responsive RCI peptide

106695	Sulfite oxidase, molybdopterin-binding component
76601	Sulfite oxidase, molybdopterin-binding component
123992	swollenin
70949	Thiamine pyrophosphate-requiring enzyme / benzoyl formate decarboxylase
103034	transcription factor 1-beta (cutinase)
2211	Transketolase
109276	Tripeptidyl peptidase II
80654	UDP-glucuronosyl and UDP-glucosyl transferase
107494	unique protein
123777	unique protein
124338	unique protein
123962	unique protein
120926	unique protein
103145	unique protein
105514	unique protein
120504	unique protein
121396	unique protein
122614	unique protein
111146	unique protein
121136	unique protein
110440	unique protein
70972	unique protein
105406	unique protein
124296	unique protein
105722	unique protein, HGT
105844	unique protein, secreted
74214	unknown protein
60422	unknown protein
112014	unknown protein
66092	unknown protein, only in fungi

124022	unknown protein
121416	unknown protein
68574	unknown protein
122089	unknown protein
111362	unknown protein
105287	unknown protein
109925	unknown protein
70894	unknown protein
60370	unknown protein
46764	unknown protein
104272	unknown protein
104295	unknown protein
124198	unknown protein
44967	unknown protein
60616	unknown protein
69375	unknown protein
119619	unknown protein
104174	unknown protein
60560	unknown protein
105237	unknown protein
60445	unknown protein
105455	unknown protein
109249	unknown protein
121439	unknown protein
121163	unknown protein
2033	unknown protein
123888	unknown protein
63868	unknown protein
103133	unknown protein
103031	unknown protein

123697	Unknown protein
54048	unknown protein
103576	unknown protein
42848	unknown protein
61526	unknown protein
75027	unknown protein
122792	unknown protein
82260	unknown protein
106686	unknown protein
58910	unknown protein
120623	unknown protein
4952	unknown protein
56853	unknown protein
61504	unknown protein
103060	unknown protein
23240	unknown protein
45598	unknown protein
103059	unknown protein
4851	unknown protein
79222	unknown protein
121486	unknown protein
105533	unknown protein
107853	unknown protein
58639	unknown protein
121653	unknown protein
81659	unknown protein
23228	unknown protein Duf1479
66696	unknown protein, Duf636
67133	unknown protein, 1TM
69483	unknown protein, 2 TM

82374	unknown protein, 3TM
50996	unknown protein, C2 domain
4626	unknown protein, C2 domain
110891	unknown protein, cupin region
54761	unknown protein, cupin region
5359	unknown protein, Duf1348
60810	unknown protein, GPR1/FUN34/yaaH protein, 6TMs
112239	unknown protein, secreted
33827	unknown protein, secreted
124282	unknown protein, SET and MYND domain
53824	unknown secreted protein
44965	zinc dependent alcohol dehydrogenase
65097	Zinc-binding oxidoreductase
23292	Zinc-binding oxidoreductase
112539	Zn2Cys6 transcription factor
120715	Zn2Cys6 transcription factor
70197	Zn-dependent hydrolase (beta-lactamase superfamily)

*strain CPK3793 was used; sporulation-upregulated genes were those described by Metz et al. (2011)

Supplementary Table S3. LaeA/LAE1 protein sequences used to construct the phylogenetic tree and identify the *T. reesei* LAE1 orthologue

Fungal species	Protein ID*	Source**
Aspergillus fumigatus	XP_752835.1	http://www.ncbi.nlm.nih.gov/protein
Neosartorya fischeri	XP_001264291.1	http://www.ncbi.nlm.nih.gov/protein
Aspergillus clavatus	XP_001268793.1	http://www.ncbi.nlm.nih.gov/protein
Aspergillus parasiticus	AAX68414.1	http://www.ncbi.nlm.nih.gov/protein
Aspergillus flavus	AAX68412.1	http://www.ncbi.nlm.nih.gov/protein
Aspergillus nidulans	AAQ95166.1	http://www.ncbi.nlm.nih.gov/protein
Monascus pilosus	ABA87010.1	http://www.ncbi.nlm.nih.gov/protein
Aspergillus niger	XP_001389674.1	http://www.ncbi.nlm.nih.gov/protein
Aspergillus oryzae	XP_001819665.1	http://www.ncbi.nlm.nih.gov/protein
Aspergillus terreus	XP_001210764.1	http://www.ncbi.nlm.nih.gov/protein
Coccidioides immitis	XP_001243806.1	http://www.ncbi.nlm.nih.gov/protein
Pyrenophora tritici-repentis	XP_001934837.1	http://www.ncbi.nlm.nih.gov/protein
Phaeosphaeria nodorum	XP_001801609.1	http://www.ncbi.nlm.nih.gov/protein
Cochliobolus heterostrophus	91933	http://genome.jgi-psf.org/
Neurospora crassa	XP_965786	http://www.ncbi.nlm.nih.gov/protein
Neurospora discreta	96073	http://genome.jgi-psf.org/
Neurospora tetrasperma	38437	http://genome.jgi-psf.org/
Magnaporthe grisea	MGG_01233.6	http://www.broadinstitute.org/annotation/genome
Chaetomium globosum	CHGG_01690.1	http://www.broadinstitute.org/annotation/genome
Fusarium graminearum	FGSG_00657.3	http://www.broadinstitute.org/annotation/genome
Fusarium verticillioides	FVEG_00539.3	http://www.broadinstitute.org/annotation/genome
*Fusarium oxysporum 1****	FOXG_00975.2	http://www.broadinstitute.org/annotation/genome
*F. oxysporum 2****	FOXG_14806.2	http://www.broadinstitute.org/annotation/genome
Nectria haematococca	90349	http://genome.jgi-psf.org/
Trichoderma atroviride	42103	http://genome.jgi-psf.org/
Trichoderma virens	31676	http://genome.jgi-psf.org/

Trichoderma reesei	41617	http://genome.jgi-psf.org/

* protein IDs are preferentially given as those used in the NCBI database; when proteins are only present in genome databases, the respective protein ID used there is given

** wherever possible, the NCBI database was given priority; otherwise the respective genome databases are given

*** *F. oxysporum* contains 2 paralogues of LAE1, neither of which could be excluded and therefore both of them were used in the phylogenetic tree

Supplementary Table S4. Oligonucleotide primers used in thus study

Name	*Sequence (5'-3')
lae1 gene deletion	
5Trlae1Hind	TAAGCTTCACTCGCTTGTGTCTTC
5Trlae1Xho	TCTCGAGCGTTTATAGTGAGTAATGGC
3Trlae1Xho	TCTCGAGCTATTGCACTCTGTAAGCC
3Trlae1Apa	TGGGCCCTGGGTAGTGTTTCGTAATG
tef1-lae1 construction	
tef1Xhofw	GCCTCGAGGGACAGAATGTAC
ClaSalrv	AGTCGACATCGATGACGGTTTGTGTGATGTAGCGTG
TrLae1ATGCla	GCTATCGATGTCTCGAAACGCTCCCAAC
TrLae1TermHind	CGAAGCTTGCCCAAGGTCATCTTTCATTG
cDNA amplification	
LAE1ATG	GCCATGTCTCGAAACGCTC
LAE1stop	GCTTACAGAGTGCAATAGGC

*Respective restriction sites are underlined

Supplementary Table S5. Primers for cellulase and *lae1* transcript quantification by Real Time PCR

Gene	Forward Primer (5' to 3')	Reverse Primer (5' to 3')
tef1 *	CCACATTGCCTGCAAGTTCGC	GTCGGTGAAAGCCTCAACGCAC
cel7a (cbh1)	CCGAGCTTGGTAGTTACTCTG	GGTAGCCTTCTTGAACTGAGT
cel6a (*cbh2*)	ACTACAACGGGTGGAACATTAC	CGTGGATGTACAGCTTCTCG
lae1	ACTGGAGATTGACTGGATGC	TTCTGCGTCTGGTAGCCTC

* *tef1* was used as a reference gene
